# Pool water quality and prevalence of microbes in filter backwash from metro-Atlanta swimming pools

**DOI:** 10.2166/wh.2017.150

**Published:** 2018-02

**Authors:** Jennifer L. Murphy, Michele C. Hlavsa, Brittany C. Carter, Candace Miller, Narayanan Jothikumar, Taryn R. Gerth, Michael J. Beach, Vincent R. Hill

**Affiliations:** Waterborne Disease Prevention Branch, Centers for Disease Control and Prevention, 1600 Clifton Road NE, Mailstop C-09, Atlanta, GA 30329, USA

**Keywords:** *Cryptosporidium* spp., *Escherichia coli*, filter backwash, *Giardia duodenalis*, *Pseudomonas aeruginosa*, swimming pools

## Abstract

During the 2012 summer swim season, aquatic venue data and filter backwash samples were collected from 127 metro-Atlanta pools. Last-recorded water chemistry measures indicated 98% (157/161) of samples were from pools with ≥1 mg/L residual chlorine without stabilized chlorine or ≥2 mg/L with stabilized chlorine and 89% (144/161) had pH readings 7.2–7.8. These water quality parameters are consistent with the 2016 Model Aquatic Health Code (2nd edition) recommendations. We used previously validated real-time polymerase chain reaction assays for detection of seven enteric microbes, including *Escherichia coli*, and *Pseudomonas aeruginosa. E. coli* was detected in 58% (93/161) of samples, signifying that swimmers likely introduced fecal material into pool water. *P. aeruginosa* was detected in 59% (95/161) of samples, indicating contamination from swimmers or biofilm growth on surfaces. *Cryptosporidium* spp. and *Giardia duodenalis* were each detected in approximately 1% of samples. These findings indicate the need for aquatics staff, state and local environmental health practitioners, and swimmers to each take steps to minimize the risk of transmission of infectious pathogens.

## INTRODUCTION

The incidence of outbreaks associated with use of aquatic venues, such as hot tubs/spas, swimming pools, and interactive water play venues, has significantly increased since 1978 (Hlavsa *et al*. 2015). In the 2016 Model Aquatic Health Code (MAHC; http://www.cdc.gov/mahc/editions/current.html), the US Centers for Disease Control and Prevention (CDC) recommends maintaining the water pH at 7.2–7.8 and residual chlorine concentration at 1–10 mg/L in aquatic venues (other than hot tubs/spas) not using stabilized chlorine, or 2–10 mg/L when using stabilized chlorine. While the extremely chlorine-tolerant *Cryptosporidium* spp. can survive for up to 7.3–10.6 days in water containing 1 mg/L of residual chlorine (Shields *et al*. 2008a; Murphy *et al*. 2015), most bacteria and viruses are inactivated within minutes.

In 2011–2012, 69 US outbreaks (77% of all recreational water-associated outbreaks) were associated with aquatic venues, resulting in at least 1,309 cases and 73 hospitalizations. While chlorine is an effective and efficient barrier to the transmission of most infectious pathogens in aquatic venues, approximately one-third of the 69 outbreaks were suspected or confirmed to be caused by chlorine-susceptible infectious pathogens (Hlavsa *et al*. 2015). Almost one in eight (11.9% (7,662/64,580)) routine inspections conducted in 16 local jurisdictions in 2013 identified disinfectant concentration violations (Hlavsa *et al*. 2016).

Backwashing, a procedure routinely performed as particulates accumulate in the pool filter, involves reversing the flow of water through the filter and discharging released particulates to waste. Backwash thus represents a useful sample for analysis of microbes in pool water that can accumulate in pool filters. A previous US microbiological survey of filter backwash samples from public aquatic venues focused on detection of only *Cryptosporidium* spp. and *Giardia intestinalis* (syn. *duodenalis*) (Shields *et al*. 2008a, 2008b). This study builds on the previous work by setting out to both collect aquatic venue-characterizing data, including last-recorded residual chlorine concentrations and pH, and testing filter backwash samples for evidence of seven enteric microbes and one biofilm-associated microbe (*Pseudomonas aeruginosa*).

## METHODS

### Aquatic venue data collection

During June–August 2012, a scheduled visit to a convenience sample of metro-Atlanta public pools took place and aquatic venue data were collected using a standardized form. Data included: pool volume, location (indoor versus outdoor), setting, and primary patron designation (e.g., adults and children or primarily children); type of filter media; stabilized chlorine usage; last-recorded residual chlorine concentration and pH; and estimated number of days since last filter backwash. No pool identifiers were collected.

### Backwash sample collection and processing

Filter backwash samples were collected from each pool with assistance from trained pool staff. Within 30 seconds of initiation of backwash discharge, an 800 mL sample was collected into a sterile plastic bottle containing sodium thiosulfate (0.025% (w/v)) to neutralize any residual chlorine present. Samples were stored at 4 °C and transported to the laboratory.

Samples were stored at 4 °C for 1–3 weeks, then concentrated using a polyethylene glycol precipitation (PEG) procedure (Polaczyk *et al*. 2008). Pelleted materials were re-suspended in 1 mL of phosphate buffered saline (PBS)/1% Tween 80/0.01% Antifoam A solution and transferred to a storage tube. An additional 1 mL of the PBS solution was used for rinsing. Two 0.5-mL aliquots were transferred into cryovials containing 0.5 mL 2X lysis buffer (UNEX buffer, Microbiologics, St. Cloud, MN) and stored at −80 °C.

### Backwash sample analysis

Nucleic acids were extracted from backwash concentrates using a bead beating procedure (Hill *et al*. 2010), followed by nucleic acid purification using silica spin columns (Omega Bioteck, Norcross, GA). A final purification step was performed using a Zymo-Spin^™^ IV-HRC polyvinylpolypyrrolidone (PVPP) spin column (Zymo Research Corporation, Orange, CA) and eluted into a final volume of 80 μL TE buffer.

Five-microliter volumes of nucleic acid extracts were tested using TaqMan assays targeting the following regions or genes: *Escherichia coli* uidA (Sandhya *et al*. 2008), *Cryptosporidium* spp. 18S rRNA (Jothikumar *et al*. 2008), *Giardia duodenalis* 18S rRNA (Jothikumar, manuscript in preparation. The highly conserved yet specific 18S rDNA primers (18SJVGF and 18SJVGR) and probe (18SJVGP) were designed to detect all seven (A–G) G. *duodenalis* assemblages.), *E. coli* O157:H7 *eae* (Sharma & Dean-Nystrom 2003), norovirus GI/GII ORF1-ORF2 junction (Hill et al. 2010), adenovirus Hexon (Jothikumar *et al*. 2005), and *Pseudomonas aeruginosa ecfX* (Amagliani *et al*. 2012). Real-time PCR amplifications were performed on ABI 7500 (Applied Biosystems, Foster City, CA) using conditions described previously. A sample was considered positive for an analyte when a cycle threshold (Ct) value <40 was obtained.

### Data analysis

Data were entered into a Microsoft Access (Redmond, WA, USA) database and analyzed using SAS 9.3 (Cary, NC, USA). Fisher’s exact test was used to test for significance at an alpha level of 0.05.

## RESULTS

During the 2012 summer swim season, 127 metro-Atlanta public pools were visited a total of 161 times, resulting in 161 filter backwash samples. Repeat visits were made to 30 pools. Due to the amount of time between visits (median: 49 (range: 6–59) days), each visit was considered to be an independent observation for these analyses. Pool volumes ranged from 500 to 800,000 (median: 75,000) gallons; 104 (65%) samples were from pools located outdoors and 89 (55%) from membership/club pools (Table 1). The vast majority (*n* = 145, 90%) of samples were from pools designated for both adults and children. Nearly all (*n* = 160, 99%) samples were from pools using sand filters. All pools used at least chlorine for disinfection. Nearly half (*n* = 71, 44%) of samples were from pools using stabilized chlorine; all of these were outdoor pools.

Pool water quality test results were last recorded the day of collection of 127 (82%) of 155 samples and a median of 1 day (range: 1–9 days) prior to collection of 28 samples (18%) (Note: CDC’s MAHC and most state and local codes for public aquatic venues require water quality testing at least once daily and recording the result). Last-recorded water quality measures prior to the collection of 157 (98%) samples fell within 2016 MAHC recommendations for residual chlorine concentration; this included 57 (100%) samples from indoor pools and 100 (96%) from outdoor pools. Of 101 samples from outdoor pools, 71 (70%) samples were from pools using stabilized chlorine, of which, 67 (94%) were from pools meeting the minimum residual chlorine recommendations. Of the 30 samples from outdoor pools that did not use stabilized chlorine, all met the minimum residual chlorine recommendations. Stabilized chlorine usage data were not available for three outdoor pools; however, each had a chlorine residual of ≥2 mg/L. There were no statistically significant differences in stabilized chlorine usage between pool settings (*p* = 0.388). The last-recorded pH ranged 7.2–7.8 prior to the collection of 144 (89%) samples.

Log books indicated the last backwash had been performed the day of collection of 31 (20%) of 157 samples, within 1–3 days before collection of 85 (54%) samples, and ≥4 days before collection of 41 (26%) samples. The median time since last backwash was 2 days (range: 0–14 days). Overall, 121 (75%) backwash samples were PCR-positive for at least one of the assayed microbes; 93 (58%) were PCR-positive for at least one of the assayed enteric microbes. Two samples (1.2%) were PCR-positive for *G. duodenalis* and one sample (0.6%) was PCR-positive for *Cryptosporidium* spp.; each of these three samples was also positive for *E. coli*. None of the 161 samples were PCR-positive for *E. coli* O157:H7, norovirus GI, norovirus GII, or adenovirus. Of 161 samples, 95 (59%) were PCR-positive for *P. aeruginosa*.

The proportion of backwash samples positive for *E. coli* significantly differed (*p* = 0.048) between membership/club (49%) and municipal (70%) pools (Table 1). There were no statistically significant differences in the proportion of backwash samples that tested positive for *E. coli* or *P. aeruginosa* by location, primary patron designation, stabilized chlorine usage, time since last backwash, or whether the last backwash occurred on a weekend or a week day.

## DISCUSSION

During the 2012 summer swim season, a convenience sample of metro-Atlanta public pools were visited and aquatic venue data and filter backwash samples were collected (CDC 2013). The data from this survey indicate that introduction of feces into the swimming pools was relatively common (with 58% of samples positive for *E. coli*), which suggests that enteric pathogens could also be introduced. The proportion of samples positive for *E. coli* was significantly lower in membership/club pools than in municipal pools, which might reflect differences in knowledge and practices related to swimmer hygiene.

*P. aeruginosa* was detected in 59% of backwash samples collected. Aquatic venue water is often contaminated with this pathogen as it can rapidly accumulate in biofilm where it is protected from disinfectants such as chlorine (Rice *et al*. 2012). Additionally, organic load (e.g., environmental particulates, sunscreen) in aquatic venues can protect microbes from disinfection, reduce disinfectant concentration, or serve as nutrients to support microbial growth.

*Cryptosporidium* is the leading cause of US gastroenteritis outbreaks associated with aquatic venues (Hlavsa *et al*. 2015), largely due to its extreme tolerance to chlorine. In this study, approximately 1% of backwash samples were PCR-positive for *Cryptosporidium* spp. and approximately 1% were PCR-positive for moderately chlorine-tolerant *G. duodenalis*. A previous US survey found multiple backwash samples positive for *Cryptosporidium* or *Giardia* (Shields *et al*. 2008b). While no outbreaks associated with survey pools were detected during the 2012 swim season, these findings highlight the potential for fecal introduction of chlorine-tolerant pathogens and the subsequent risk of pathogen transmission and outbreaks. This risk might be increased if stabilized chlorine is utilized for treatment, as it substantially delays chlorine inactivation of *Cryptosporidium* oocysts (Murphy *et al*. 2015). Inclusion of secondary disinfection systems (e.g., ultraviolet light or ozone) to increased-risk aquatic venues, such as wading pools and interactive water play venues, is recommended in the MAHC.

This study was subject to multiple limitations. First, the pools sampled were a convenience sample; therefore, study findings cannot be generalized to pools in metro-Atlanta or beyond. However, acute gastrointestinal illness outbreaks associated with aquatic venues occur across the United States; this suggests bathers introduce feces and pathogens to aquatic venue water. Second, last-recorded water chemistries are a one-time representation of the state of the operation and maintenance of a given pool and might not truly reflect overall operation and maintenance. Third, pool temperature data were not recorded; these data could help characterize environmental conditions that might promote biofilm growth. Fourth, molecular testing results alone cannot be used to determine whether the detected pathogens were viable or infectious, or thus determine swimmer risk. Fifth, PCR is unable to detect DNA or RNA that has been damaged by chlorine, which might result in an underestimate of microbial prevalence.

## CONCLUSION

This study found that 98% of samples were from pools with water quality readings consistent with CDC’s residual chlorine recommendations and 89% with pH within the recommended range. Molecular tests indicated fecal contamination was present in 58% of backwash samples and underscore the need to reduce introduction of fecal contaminants into pools by swimmers and maintain appropriate water quality measures to prevent transmission of infectious pathogens that are introduced. Additionally, 59% of backwash samples were positive for *P. aeruginosa*, indicating contamination from swimmers or biofilm growth on pool system surfaces. These findings identify the need for: (1) aquatics staff to maintain adequate disinfectant concentration to inactivate infectious pathogens and to routinely clean surfaces to control microbial growth in pools; (2) state and local environmental health practitioners and pool operator training organizations to continue to educate aquatic venue operators about how to properly operate and maintain public aquatic venues and to enforce such standards through inspections; and (3) swimmers, with guidance provided by pool operators and public health practitioners, to take steps to help prevent introduction of pathogens (e.g., not swimming when ill with diarrhea and showering before entering the water).

## Figures and Tables

**Table 1 |:** Microbes in filter backwash samples (*n* = 161) from metro-Atlanta pools, by select characteristics

Characteristic	PCR positive for *E. coli* (*N* = 93)^a^ *n* (%)	*p*-value^b^	PCR positive for *P. aeruginosa* (*N* = 95)^a^ *n* (%)	*p*-value^b^
Location				
Outdoor (*n* = 104)	60 (58)	1.000	67 (64)	0.067
Indoor (*n* = 57)	33 (58)	Ref.	28 (49)	Ref.
Setting				
Municipal (*n* = 37)	26 (70)	**0.048**	22 (59)	0.843
Waterpark (*n* = 35)	23 (66)	0.114	18 (51)	0.316
Membership/Club (*n* = 89)	44 (49)	Ref.	55 (62)	Ref.
Primary patron designation				
Primarily children (*n* = 15)	11 (73)	0.274	10 (67)	0.595
Adults and children (*n* = 145)	81 (56)	Ref.	85 (59)	Ref.
Stabilized chlorine usage				
No (*n* = 90)	53 (59)	0.751	51 (57)	0.522
Yes (*n* = 71)	40 (56)	Ref.	44 (62)	Ref.
Time since last backwash				
1–3 days (*n* = 85)	44 (52)	0.090	47 (55)	0.673
≥ 4 days (*n* = 41)	24 (59)	0.328	27 (66)	0.805
Same day (*n* = 31)	22 (71)	Ref.	19 (61)	Ref.
Day of last backwash				
Weekday (*n* = 138)	77 (56)	0.333	82 (59)	1.000
Weekend (*n* = 19)	13 (68)	Ref.	11 (58)	Ref.

*Note*: Denominators might vary slightly due to missing values.

a Cycle threshold (Ct) value <40 was considered a positive result.

b Fisher’s exact test.

## References

[R1] Amagliani G, Parlani ML, Brandi G, Sebastianelli G, Stocchi V & Schiavano GF 2012 Molecular detection of *Pseudomonas aeruginosa* in recreational water. Int. J. Environ. Health Res 22, 60–70. 21671204 10.1080/09603123.2011.588325

[R2] CDC 2013 Microbes in pool filter backwash as evidence of the need for improved swimmer hygiene – Metro-Atlanta, Georgia, 2012. MMWR 62 (19), 385–388. 23677046 PMC4604905

[R3] Hill VR, Mull B, Jothikumar N, Ferdinand K & Vinje J 2010 Detection of GI and GII noroviruses in ground water using ultrafiltration and TaqMan real-time RT-PCR. Food Environ. Virol 2, 218–224.

[R4] Hlavsa MC, Roberts VA, Kahler AM, Hilborn E, Mecher T, Beach M, Wade T & Yoder J 2015 Outbreaks of illness associated with recreational water – United States, 2011–2012. MMWR 64 (24), 668–672. 26110837 PMC4584744

[R5] Hlavsa MC, Gerth TR, Collier SA, Dunbar EL, Rao G, Epperson G, Bramlett B, Ludwig DF, Gomez D, Stansbury MM, Miller F, Warren J, Nichol J, Bowman H, Huynh BA, Loewe KM, Vincent B, Tarrier AL, Shay T, Wright R, Brown AC, Kunz JM, Fullerton KE, Cope JR & Beach MJ 2016 Immediate closures and violations identified during routine inspections of public aquatic facilities – Network for Aquatic Facility Inspection Surveillance, Five States, 2013. MMWR Surveill. Summ 65 (SS-5), 1–26. 10.15585/mmwr.ss6505a1 27199095

[R6] Jothikumar N, Cromeans TL, Hill VR, Lu X, Sobsey MD & Erdman DD 2005 Quantitative real-time PCR assays for detection of human adenoviruses and identification of serotypes 40 and 41. Appl. Environ. Microbiol 71, 3131–3136. 15933012 10.1128/AEM.71.6.3131-3136.2005 PMC1151802

[R7] Jothikumar N, da Silva AJ, Moura I, Qvarnstrom Y & Hill VR 2008 Detection and differentiation of *Cryptosporidium hominis* and *Cryptosprodium parvum* by dual TaqMan assays. J. Med. Microbiol 57, 1099–1105. 18719179 10.1099/jmm.0.2008/001461-0

[R8] Murphy JL, Arrowood MJ, Lu X, Hlavsa MC, Beach MJ & Hill VR 2015 Effect of cyanuric acid on the inactivation of *Cryptosporidium parvum* under hyperchlorination conditions. Environ. Sci. Technol 49, 7348–7355. 26042636 10.1021/acs.est.5b00962 PMC10919751

[R9] Polaczyk AL, Narayanan J, Cromeans TL, Hahn D, Roberts JM, Amburgey JE & Hill VR 2008 Ultrafiltration-based techniques for rapid and simultaneous concentration of multiple microbe classes from 100-L tap water samples. J. Microbiol. Methods 73, 92–99. 18395278 10.1016/j.mimet.2008.02.014

[R10] Rice SA, van den Akker B, Pomati F & Roser D 2012 A risk assessment of Pseudomonas aeruginosa in swimming pools: a review. J. Water Health 10 (2), 181–196. 22717744 10.2166/wh.2012.020

[R11] Sandhya S, Chen W & Mulchandani A 2008 Molecular beacons: a real-time polymerase chain reaction assay for detecting Escherichia coli from fresh produce and water. Anal. Chim. Acta 614, 208–212. 18420053 10.1016/j.aca.2008.03.026

[R12] Sharma VK & Dean-Nystrom EA 2003 Detection of enterohemorrhagic *E. coli* o157:H7 by using a multiplex real-time PCR assay for genes encoding intimin and Shiga toxins. Vet. Microbiol 93, 247–260. 12695048 10.1016/s0378-1135(03)00039-7

[R13] Shields JM, Hill VR, Arrowood MJ & Beach MJ 2008a Inactivation of *Cryptosporidium parvum* under chlorinated recreational water conditions. J. Water Health 6, 513–520. 18401116 10.2166/wh.2008.068

[R14] Shields JM, Gleim ER & Beach MJ 2008b Prevalence of *Cryptosporidium* spp. and *Giardia intestinalis* in swimming pools, Atlanta, Georgia. Emerg. Infect. Dis 14 (6), 948–950. 18507911 10.3201/eid1406.071495 PMC2600305

